# A framework for analysing learning health systems: Are we removing the most impactful barriers?

**DOI:** 10.1002/lrh2.10189

**Published:** 2019-03-21

**Authors:** Scott McLachlan, Kudakwashe Dube, Owen Johnson, Derek Buchanan, Henry W.W. Potts, Thomas Gallagher, Norman Fenton

**Affiliations:** ^1^ Electrical Engineering and Computer Science Queen Mary University of London London UK; ^2^ Fundamental Sciences Massey University Palmerston North New Zealand; ^3^ School of Computing University of Leeds Leeds UK; ^4^ Institute of Health Informatics University College London London UK; ^5^ Missoula College University of Montana Missoula Montana

**Keywords:** electronic health records, learning health systems, learning health care systems

## Abstract

**Introduction:**

Learning health systems (LHS) are one of the major computing advances in health care. However, no prior research has systematically analysed barriers and facilitators for LHS. This paper presents an investigation into the barriers, benefits, and facilitating factors for LHS in order to create a basis for their successful implementation and adoption.

**Methods:**

First, the ITPOSMO‐BBF framework was developed based on the established ITPOSMO (information, technology, processes, objectives, staffing, management, and other factors) framework, extending it for analysing barriers, benefits, and facilitators. Second, the new framework was applied to LHS.

**Results:**

We found that LHS shares similar barriers and facilitators with electronic health records (EHR); in particular, most facilitator effort in implementing EHR and LHS goes towards barriers categorised as *human factors*, even though they were seen to carry fewer benefits. Barriers whose resolution would bring significant benefits in safety, quality, and health outcomes remain.

LHS envisage constant generation of new clinical knowledge and practice based on the central role of collections of EHR. Once LHS are constructed and operational, they trigger new data streams into the EHR. So LHS and EHR have a symbiotic relationship. The implementation and adoption of EHRs have proved and continues to prove challenging, and there are many lessons for LHS arising from these challenges.

**Conclusions:**

Successful adoption of LHS should take account of the framework proposed in this paper, especially with respect to its focus on removing barriers that have the most impact.

## 1 INTRODUCTION

Learning health systems (LHS) were developed as a vehicle to advance clinical safety and health research and improve patient‐centred care, with the added goal to more fully realise the benefits and potential of electronic health records (EHR).^1^, ^2^, ^3^ The learning component of LHS can occur at multiple levels, including the personal level for individual actors (when educating doctors, patients, caregivers, etc), the team and organisational level (when revising work practice and care pathways), and at the whole system level (when the LHS demonstrates holistic learning). This work primarily focuses on learning that uses knowledge derived from collections of EHR as a digital support system to introduce programmed improvements (when introducing new workflow or decision support) or as automated learning (as promised by the introduction of AI within clinical systems).

Our exploration of LHS began by discovering that much work describing LHS is not actually identified as such within the LHS domain: Something we described as the *research community awareness challenge*.^4^ To aid researchers in appropriately identifying their works within the domain, this research group provided a taxonomy describing the nine types of LHS commonly observed in the literature and a unifying framework showing how each type positions within the larger learning health organisation.^1^ In this work, we focus on the notion of *barriers*, *benefits*, and *facilitators*, their identification, impact, and application. *Barriers* are those things that inhibit implementation and use of a particular technology or system, such as health information systems (HIS) and LHS. *Benefits* are the positive outcomes realised by resolving a barrier through engaging a facilitator. *Facilitators* are those interventions described as easing the burden of implementation and use of a technology such as EHR. A facilitator is targeted towards resolving one or more related barriers.

EHR are the enabling technology for LHS. Considerable research has consolidated knowledge on barriers, benefits, and facilitators for EHR implementations.^5^, ^6^, ^7^ However, in the LHS domain, the picture is considerably different. We believe a gap exists in the research literature in that with the exception of passing reference to LHS barriers, no such consolidation of knowledge on barriers and benefits for LHS adoption could be found. There is reference to a link between EHR barriers and LHS barriers^8^ albeit without any analytical framework. It is necessary to develop approaches for analysis and mitigation of barriers in order to facilitate LHS, just as LHS should benefit patients through the conduct of more precise, individualised medical practice.^1^ This paper presents a literature review used to close the research gap and develop such an analysis framework. The main focus of this work is therefore the development and application of that framework for use in analysing and consolidating barriers and facilitators that may be encountered in LHS implementations and determining whether benefits already identified from implementations of EHR are similar to those that may result from implementing LHS.

In the information systems literature, one often observed and widely used framework for evaluating implementation challenges is ITPOSMO. ITPOSMO identifies seven dimensions for exploring the gap between a system's design and the reality of its implementation: information, technology, processes, objectives, staffing, management, and other factors.^9^ The ITPOSMO framework was originally proposed for evaluating e‐government projects, but it has since been used to evaluate EHR projects and help explain why health information systems succeed or fail.^10^ Our work extends the ITPOSMO framework to support the comparative analysis of barriers, benefits, and facilitators for both EHR and LHS. This led to a new framework, which we call ITPOSMO‐BBF, that was then used to explore the literature on barriers, benefits, and facilitators for LHS.

Hence, this paper presents results of an investigation into those things that hinder or enable technology use in health care environments. In particular, it presents a framework for classifying and analysing barriers and facilitators and contrasting facilitators with the degree of benefit authors have ascribed to them. One of our key objectives is to help those implementing LHS to identify their own major barriers and optimise their efforts with facilitators that will not only address those barriers but whose impact will maximise implementation success.

The rest of this paper is organised as follows: Section 2 introduces the concept of LHS and presents the background and context of the research problem. Section 3 presents the methodology, particularly the framework developed for addressing the problem. The results of applying that method to the literature are presented in Section 4, along with discussion, before we conclude the paper.

## 2 LEARNING HEALTH SYSTEMS

EHR are repositories of retrospective, current, and prospective patient data stored in digital form, with the intention of supporting efficient, quality health care service delivery.^11^ Those implementing EHR have long complained of slow adoption and limited implementation success rates.^12^, ^13^, ^14^, ^15^ LHS represents a vision to transform health care.^16^, ^17^ This vision includes leveraging recent and ongoing developments in EHRs by developing new knowledge from the ever‐increasing amounts of digital routine health data accumulating within them^17^, ^18^ and innovating learning from the slower population‐based processes of evidence‐based medicine (EBM), using rapid identification of new knowledge to deliver precision medicine.^17^, ^19^ Large collections of EHR are the fuel for LHS, giving statistical power to population level insights, and the EHR also provides the delivery mechanism for decision support tools that allow clinicians to diagnose and tailor treatment decisions using patient‐level data in real time.^18^ While EHR have existed in some form for more than 40 years, LHS have existed for less than one quarter of that, and the bulk of LHS research and development has only occurred since 2011.^1^

EHR have become almost ubiquitous in health care, yet many hospitals and clinics in these countries still employ a mixture of paper and electronic records.^20^, ^21^, ^22^, ^23^ Among those EHR implementations in the hospital setting, many offer only limited functionality and occur as isolated islands of information, with separate EHRs tied to a particular ward, medical specialty, or care pathway.^24^ There have been some spectacular failures to realise the initial promise of EHRs, as with the UK's National Program for IT wherein the NHS failed to deliver effective national hospital EHRs.^25^ The design of many EHRs, particularly those from the United States, comes from health service billing software that can conflict with the needs of clinicians leading to both workflow and information presentation challenges in clinical use.^26^ The data within medical records typically exist within a specific context, and there are some limitations on transferring it to another context, or work needed to support that transfer.^27^ Many commercial EHR solutions have proprietary and cost barriers to integration with other systems and sources of health data relevant to the individual patient.^28^, ^29^ With the best intentions, many health care organisations have self‐inflicted these issues by layering inflexible new technology over existing processes and procedures in the belief that EHR implementation meant simply replacing paper records with electronic systems.^23^, ^30^ The result too often has been implementations of EHR that have failed to improve quality of care, increase efficiency, or reduce health care costs.^31^, ^32^ Behind this is a lack of understanding about how clinicians interact with computers and disagreement as to whether such interaction enables or inhibits patient‐centred care.^33^ Successful implementation of new EHRs requires clinician‐ and user‐led processes that re‐evaluate practices and procedures, with a requisite period of adaption and training for those who will use the resulting combination of new IT systems, documentation procedures, and clinical workflows.^34^, ^35^

There is growing recognition that LHS can exist at different scales from department to organisation and across multiple organisational boundaries,^1^, ^2^ but in all cases, they are limited by the functionality, quality, and interoperability of their underlying EHRs.^1^, ^36^, ^37^, ^38^ Our research is motivated by the belief that an understanding of EHR barriers, benefits, and facilitators is essential for those implementing LHS because a successful EHR is a pre‐requisite and because the challenges faced in EHR implementation are symptomatic of a range of deeper issues that impact on any major innovation in digital health.

## 3 METHOD

Our literature review followed the systematic method for identifying benefits and barriers as described in Yao et al.^39^ The method is divided into three phases: *Search and selection*, *Categorisation*, and *Analysis*.

### 3.1 Search and selection

There were two parts to the literature search. For EHRs, an initial search used the search terms (“Electronic Healthcare Record” + “Barriers” + “Benefits” + “Facilitating Factors”). A second search replaced “Healthcare” with “Health” while preserving the remaining search terms. We used a combined search engine drawing on the following repositories: Scopia, Science Direct, PubMed, EBSCOhost, DOAJ, and Elsevier.

Articles were included where they presented a scoping or systematic review of EHR implementations, providing analysis and discussion of all three elements: barriers, benefits, and facilitators. Those not meeting these requirements were rejected. For alignment with our LHS literature review,^1^ articles more than 10 years old were also rejected.

For the literature on LHS, we used the search and inclusion criteria described in our previous paper,^1^ which used the plain language search terms (“LHS” and “learning healthcare systems”) to identify works that presented or proposed a solution self‐identified within the LHS domain.

### 3.2 Categorisation

**Figure 1:**
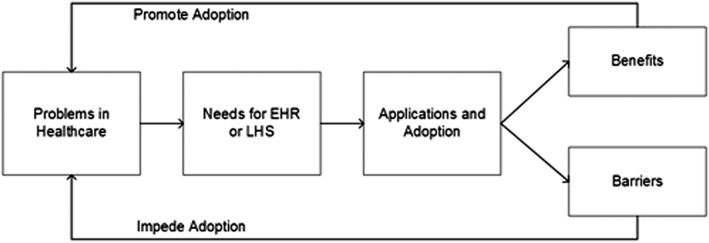
Research framework

Collected literature was divided into two sets: those providing statistical metrics for barriers and benefits and those that did not. The analysis framework was adapted from the method used in Greenhalgh et al^24^ and is shown in Figure 1. Content Analysis and Thematic Analysis^40^ and Formal Concept Analysis^41^ were used to identify and classify the barriers and benefits described from implementing EHR and LHS.

### 3.3 Analysis

**Figure 2:**
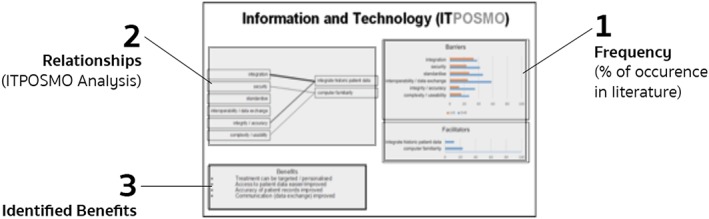
Representation of the ITPOSMO‐BBF diagram structure

Finally, we contrasted, compared, and analysed the barriers and benefits described in the EHR and LHS literature sets using an extension of the ITPOSMO methodology. This effort sought to identify similarities between facilitating factors from established EHR and LHS implementations. Precedents exist for expanding ITPOSMO to enable additional scope and functionality, including service quality analysis,^42^ survey‐based study of consumer and public perceptions,^43^ and Socratic analysis of local e‐Government.^44^ Our ITPOSMO‐BBF model used in this work adopts the aspects and dimensions of the original ITPOSMO framework^9^ and combines these with a framework for analysing barriers and facilitators from Hassan et al.^45^ The ITPOSMO framework is conventionally used to structure analysis of the gap between expectations and reality in IT projects. For our analysis, we extended ITPOSMO by adding an additional component, benefits, which may be realised directly from either mitigation of barriers or application of facilitators. This additional component, along with representation of the percentage of literature from which each barrier or facilitator was drawn, form the three sections of our ITPOSMO‐BBF diagrammatic approach shown in Figure 2.

The ITPOSMO‐BBF diagram structure (1) identifies barriers and facilitators to implementation of LHS discussed in the literature, (2) quantifies the relationships between facilitators and specific barriers, and (3) identifies benefits that authors believe will be realised when these barriers are resolved. While ITPOSMO was developed as a retrospective analysis of projects that have already completed, ITPOSMO‐BBF can be used with barriers and facilitators' data to understand, plan for, and mitigate potential barriers prior to a new implementation of LHS.

**Figure 3:**
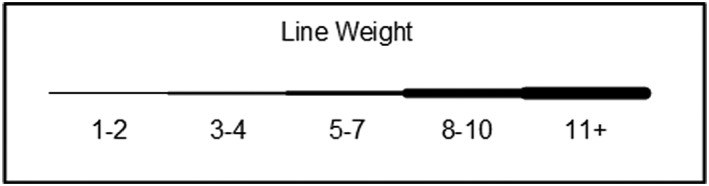
Line weights and number of papers

Each of the four ITPOSMO‐BBF diagrams in the Results section provides key data, including the percentage of EHR and LHS literature identifying an individual barrier or facilitator; the contextual relationships observed in authors' discussion of barriers and facilitators; and the corresponding benefits authors ascribe to either resolving the identified barriers or engaging with the described facilitators. The frequency of attention drawn by authors to each relationship between a barrier and facilitator is shown with a weighted line. Figure 3 identifies the relationship between the thickness or weight of each relationship line and the number of authors who identified that particular relationship.

## 4 RESULTS

### 4.1 Results of literature search

**Figure 4:**
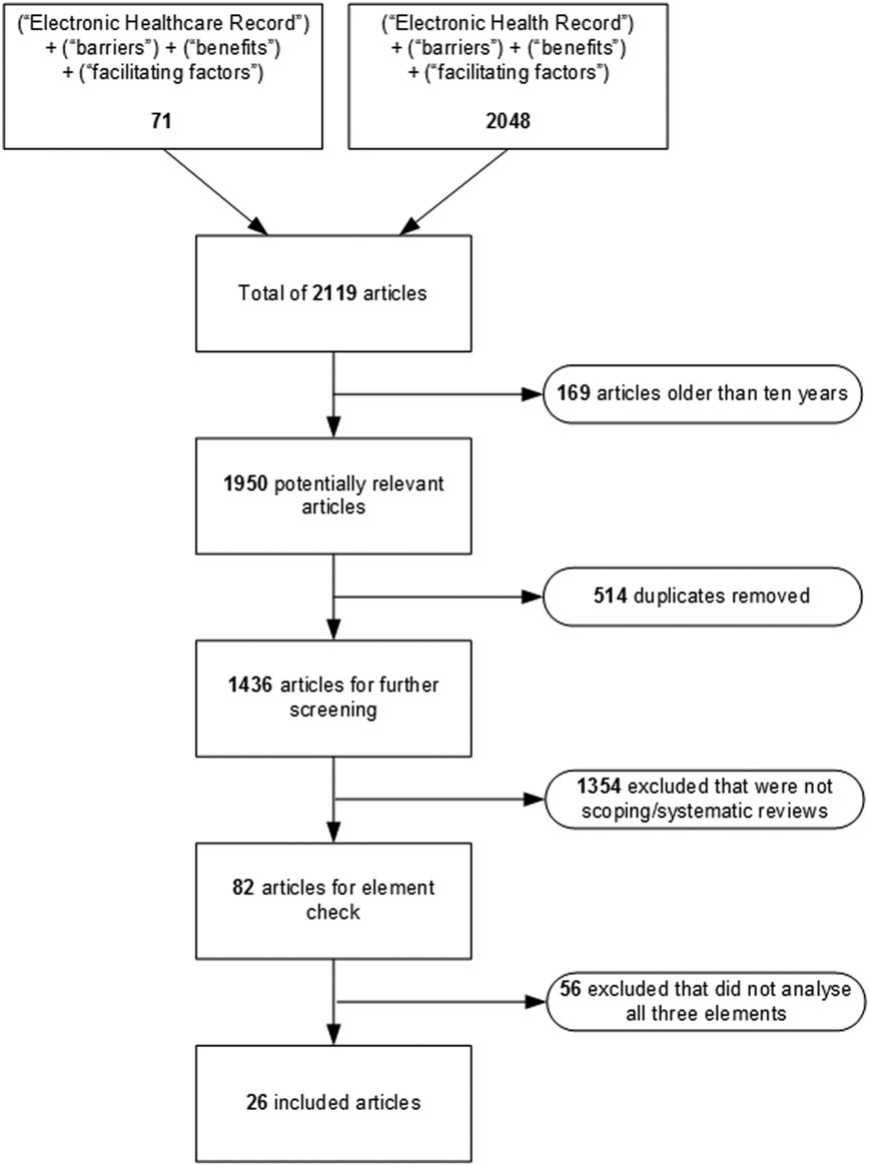
EHR literature selection

**Figure 5:**
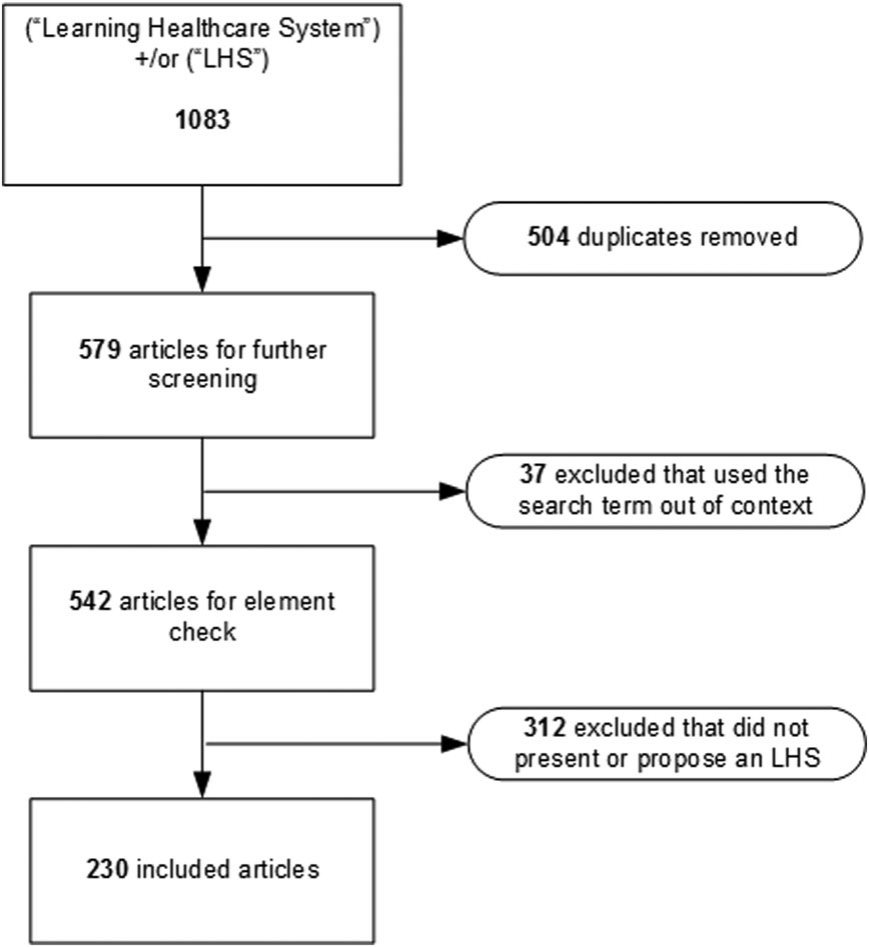
LHS literature selection

We identified 26 papers from the EHR review that, along with the 230 papers already identified in McLachlan et al,^1^ met the selection criteria. The process for resolving the literature collections for this paper is shown in Figure 4 for EHR and Figure 5 for LHS.

Of 82 scoping or systematic reviews of EHR implementations, 26 provided analysis and discussion on all three of our research elements: barriers, benefits, and facilitators. The literature for LHS is currently limited, and no scoping or systematic reviews were identified within the selected literature, so instead, we conducted a thematic review of the content of the 230 selected LHS papers linked to the three elements.

### 4.2 Thematic analysis of barriers and facilitators

Content and thematic analysis^40^ was used to identify barrier, benefit, and facilitator themes, while also examining the context in which authors described themes and the overall frequency of their use within the collected literature. It was possible to categorise many of the themes identified within an overall summary or key theme: a grouping of related *like* themes. These key themes are described in Table 1, which lists the LHS and EHR literature from which that theme was identified, and whether it was discussed by the authors in the context of a barrier inhibiting implementation or use, or if appropriately engaged, would serve as a facilitator for new implementations. For example, in Table 1, key theme 3 concerns concepts of *data standardisation*, *integration*, and *interoperability* has been encountered as a barrier with respect to implementation of LHS and EHR in 14 and 11 papers, respectively. Resolving the issues of key theme 3 is also described as a potential facilitator of LHS and EHR in eight and five papers, respectively.

**Table 1:** LHS and EHR literature categorised according to results of thematic analysis

	Key Themes	Barrier		Facilitator	
		LHS	EHR	LHS	EHR
1	Willingness, interest, or motivation to adopt new HIS and frameworks	12, 13, 46, 47, 48, 49, 50, 51, 52	7, 53, 54, 55, 56	12, 48, 57, 58, 59, 60	61
2	Training and skills with computer systems and HIS	52, 62, 63, 64, 65	66, 67	68, 69	5, 54, 70, 71, 72, 73
3	Data standardisation, interoperability, and integration	12, 13, 15, 46, 49, 65, 69, 74, 75, 76, 77, 78, 79, 80, 81	14, 15, 48, 50, 61, 64, 75, 78, 82, 83, 84	57, 74, 76, 77, 85, 86, 87, 88	3, 70, 73, 82, 89
4	Changes to legislation, policy, and government‐mandated financial factors (incentives or penalties)	12, 48, 49, 60, 61, 62, 67, 75, 87, 90, 91, 92, 93, 94, 95	12, 50, 67, 82, 84, 96	48, 61, 82, 85, 91, 93, 94, 97, 98, 99, 100	6, 14, 15, 61, 83, 86, 96, 101, 102
5	Capital investment, implementation, maintenance, and support costs	12, 49, 58, 101, 103, 104, 105, 106, 107, 108	5, 6, 15, 50, 53, 54, 55, 56, 67, 70, 71, 73, 89, 109, 110, 111	3, 13, 18, 63, 64, 66, 85, 104, 107, 112	6, 7, 50, 56, 73, 89, 109, 111
6	Impact of LHS on health outcomes and patient‐clinician encounter within the patient care workflow	46, 48, 61, 67, 75, 78, 84, 86, 101	6, 7, 53, 54, 55, 70, 71, 72, 73, 89, 109, 111	46, 77	
7	Privacy, security, data integrity, and accuracy	3, 12, 47, 48, 49, 52, 57, 61, 62, 75, 77, 78, 81, 83, 86, 92, 101, 102, 106, 113, 114	5, 7, 15, 46, 53, 54, 55, 56, 67, 70, 73, 96, 111, 115	76, 85	6, 65, 73
8	Approvals and ethics oversight for use of digital health data	64, 65, 90, 96, 101, 113, 116, 117, 118, 119, 120	78, 121	96, 101	
9	Organisational culture, management, and clinician attitudes to change	13, 17, 52, 57, 64, 65, 80, 85, 87, 94, 122	53, 54, 70, 109, 111, 115, 123	3, 69, 80, 87, 124	70, 111, 125
10	Identifying and involving all relevant stakeholders	47, 48, 49, 51, 57, 62, 68, 74, 78, 85, 87, 97, 101, 102, 106, 107, 117, 124, 126, 127, 128, 129	7, 37, 130	3, 58, 87, 105, 106, 118, 124, 131	7, 54, 55, 78, 111

**Figure 6:**
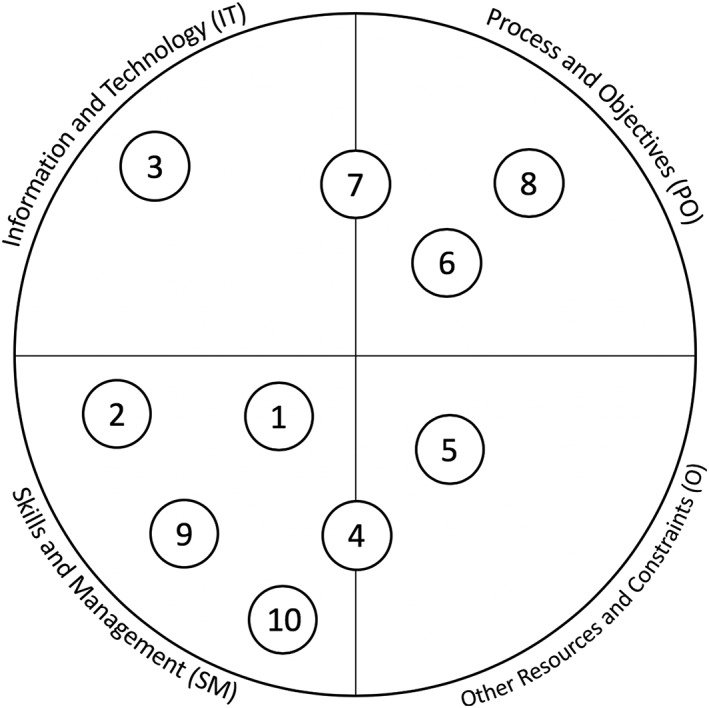
Linking key themes to ITPOSMO (numbers shown identify themes listed in Table 1)

Figure 6 shows that each of the key themes falls within one of the ITPOSMO dimensions, except for key themes 4 and 7 that fall across the boundaries of two domains. It was important to map the key themes to the ITPOSMO elements to reveal where authors were focusing their efforts and identify whether barriers existed for which limited or no mitigation through application of facilitators had occurred.

Table 1 maps the literature to the key themes that are significant with respect to barriers, benefits, and facilitators. Figure 6 then maps these key themes to the ITPOSMO elements, or domains, which form the basis of ITPOSMO‐BBF that this paper uses as its analytical framework.

### 4.3 Application of ITPOSMO‐BBF methodology

#### 4.3.1 Information and technology

**Figure 7:**
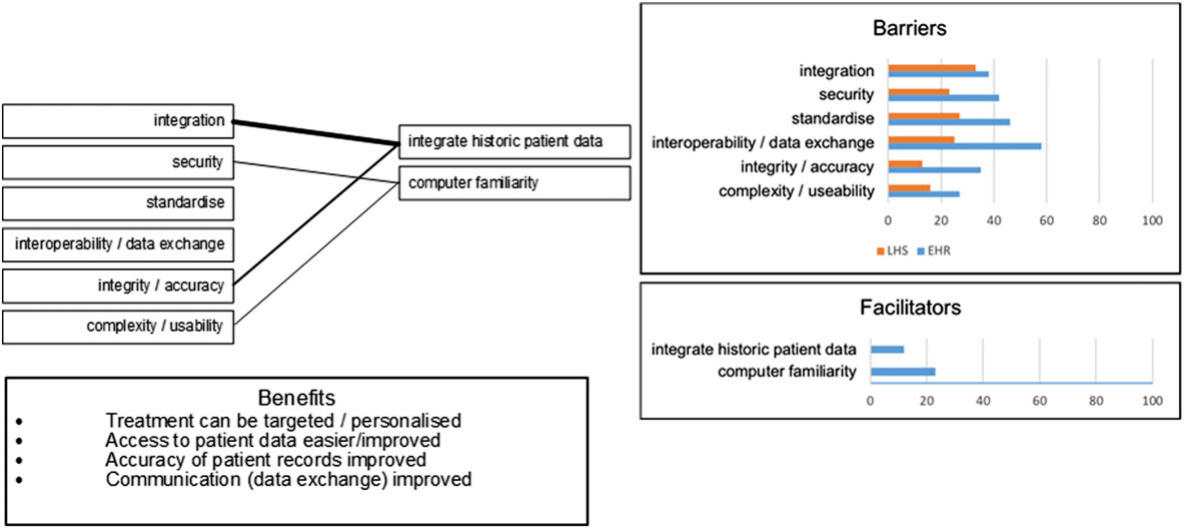
Information and technology (**IT**POSMO)

A number of information and technology barriers were described by authors as shown in Table 1. These were most often issues that arose resulting from the stand‐alone and bespoke nature of health systems, coupled with a lack of ability to combine systems or data in any simple, inexpensive, or meaningful way. Little effort has been expended in devising facilitators to resolving these barriers, even though there are important benefits that could be realised. Figure 7 shows that the most frequently discussed facilitator that authors considered would realise a number of the listed benefits was the seemingly simple act of integrating historic patient data so that any new system presented a complete picture of the patient.

#### 4.3.2 Process and objectives

**Figure 8:**
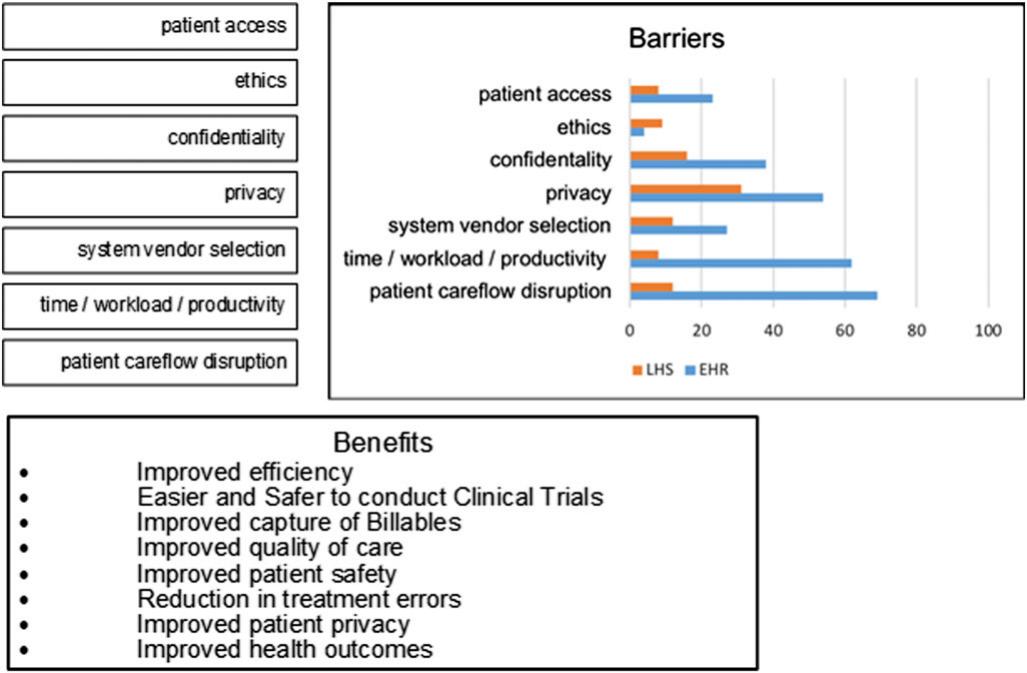
Process and objectives (IT**PO**SMO)

Many of the process and objectives (PO) barriers might reasonably appear to fall within the remit of ethicists. The small number remaining was raised by clinicians who work closest with patients, namely, nurses and general practitioners. Many of the potential benefits authors felt would result from resolving the PO barriers would appear to deal directly with issues of patient safety and confidence with health services, yet surprisingly no single facilitator was directly attributable to resolving one of the PO barriers and realising the benefits presented in Figure 8.

#### 4.3.3 Skills and management

**Figure 9:**
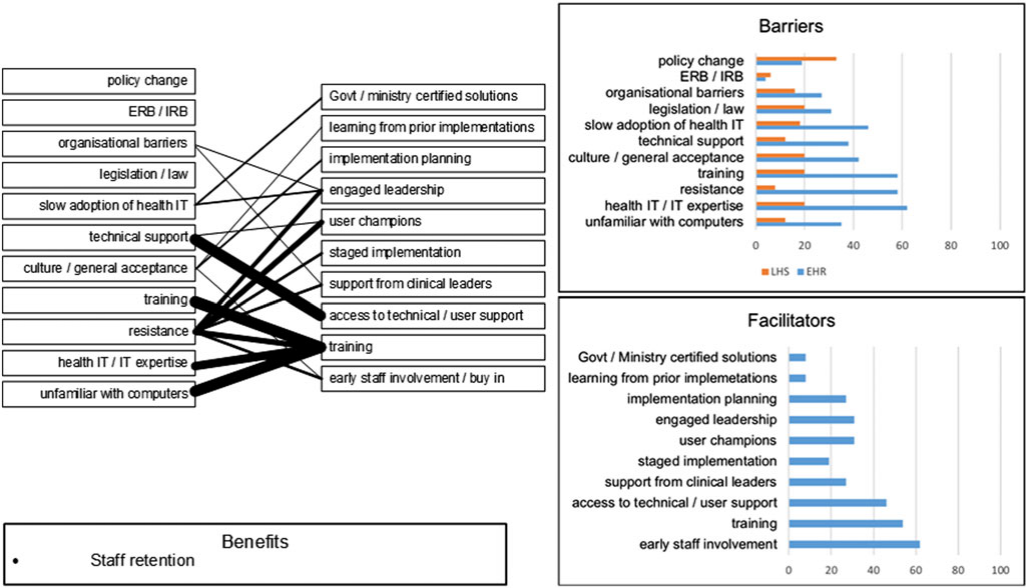
Skills and management (ITPO**SM**O)

Most effort aimed at facilitating EHR has gone towards resolving “human factor” barriers, even though the literature only makes one reference to a benefit as shown in the skills and management (SM) element of ITPOSMO in Figure 9. Even in the case of technical support and training, these were described by authors in the context of developing skills and managing staff resistance, yet no single author reported that any of these facilitators was actually reducing staff resistance to technology or improving adoption rates for EHR. While there is strong interest directed towards resolving adoption issues, the facilitators presently being employed do not appear to have substantially resolved these issues, as the EHR adoption problem persists.

#### 4.3.4 Other resources and constraints

**Figure 10:**
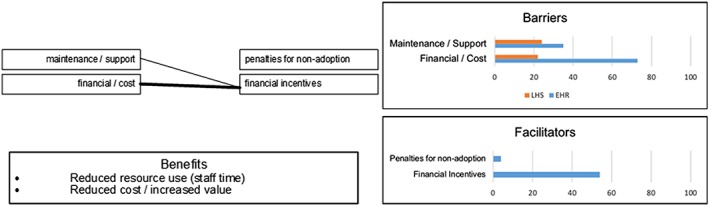
Other resources and constraints (ITPOSM**O**)

The other resources and constraints (O) element shown in Figure 10 reviews those attributes not falling within the first three ITPOSMO elements, including finance, maintenance, and user and systems support. While financial incentives that had been enshrined in the laws of countries like the United States was discussed by more than half of all EHR papers, only three mentioned the potential for penalties for non‐adoption contained in the same legislation to be a facilitator. Note, however, that none of the three led to the belief that the threat of penalties had helped an implementation of HIS.

### 4.4 Barriers

Almost every author spoke of barriers or challenges in implementing EHR and LHS. Some present as *barriers to entry*, such as the high financial burden to implement and support HIS technology^6^, ^54^, ^70^ and issues relating to complicated and inconsistent legislation.^7^, ^55^, ^56^ Others present as *barriers to success*, including the need for data and systems standardisation^7^, ^54^, ^73^, ^109^ and issues with interoperability^5^, ^7^, ^53^ or integration.^53^, ^71^ Many also describe *barriers to organisational culture*,^132^ such as clinical users and patients expressing negative attitudes towards, and reservations with, the use of computing systems, resistance to potential changes in workflow that it was believed could disrupt the flow of patient care,^6^, ^53^, ^71^ the impact on time management,^5^, ^73^, ^89^ and opposition to the need for training and staff development in HIS use.^6^, ^70^, ^71^ The potential for issues of data accuracy and integrity was said to arise from the use of HIS, with lasting impacts for patient care and health outcomes.^7^, ^54^, ^73^, ^89^ Concerns were expressed that systems producing, storing, exchanging, or amalgamating health data would negatively affect patient privacy and confidentiality,^5^, ^53^, ^70^, ^109^ with adequate data security seen as an unresolved primary challenge.^70^, ^73^, ^111^ We also identified barriers that arose from overpromised HIS technology and budget overruns that add to negative perceptions of EHR and LHS. Barriers were discussed consistently for both EHR and LHS.

### 4.5 Facilitators

Overcoming barriers has represented a significant challenge for EHR implementations, which is why organisations invest in efforts to identify and engage facilitators that will lead to successful implementations.^53^, ^70^, ^71^ Some problems are seen as endemic and specific to the IT industry,^71^ while others require change in health policy and legislation.^6^, ^53^, ^71^ The literature demonstrates that patience,^71^ committed leadership,^53^, ^109^ systematic planning, and incremental implementation^70^, ^109^ have considerably positive effects.^71^, ^109^ Early collaboration with clinical users,^53^, ^70^ provision of general computer and EHR‐specific training,^54^, ^70^, ^71^, ^109^ and engaging user champions to drive acceptance and reduce user frustration^70^, ^72^, ^111^ were substantial human factors that counter resistance. Resistance is considered significant enough factor that some medical schools mandate or recommend students complete specific training in the implementation and use of HIS.^133^, ^134^, ^135^

### 4.6 Benefits

Organisations contemplating HIS implementation do so with some intention of realising one or more benefits.^136^ While it could be argued that any benefit improves the health of patients, even indirectly, this research found that benefits fall broadly into two categories. First, there are those that have a direct positive effect on health outcomes for patients. These include any that increase patient safety,^7^, ^70^ reduce harm from treatment or medication errors,^70^, ^73^ or improve the overall quality of health care.^6^, ^54^, ^73^ Second, those seeking to improve some metric of health care delivery by increasing efficiencies and accountability,^6^, ^54^, ^70^ or reducing waste and over‐consumption of resources which accordingly increases overall capacity of the health care system.^6^, ^70^, ^109^ A key point was that while LHS are seen to significantly benefit the conduct of many types of clinical trials, EHR were not discussed by any author as doing so to any similar degree. This, in spite of the fact that EHRs are the constituent components of all LHS.

## 5 DISCUSSION

While the benefits of LHS build and significantly expand on those put forward for EHR, the barriers described for both are similar. This confirms LHS are inheriting unresolved challenges from EHR. For this reason, we chose to also investigate the factors identified as facilitating EHR implementation to assess whether it is possible that these may aid in resolving LHS implementation challenges.

### 5.1 Acceptance of EHR

A novel causal factor receiving attention is *digital disruption*. Digital disruption is a catch‐all term for a range of related issues, described as the changes facilitated by the introduction of digital technologies that occur at a pace and magnitude that disrupt established ways of value creation, social interactions, doing business, and, more generally, our way of thinking.^137^ One group in Australia have attributed the failure of more than half of all EHR system implementations to poor understanding and management of digital disruption, failure to understand and manage disruption to clinical workflows, the anxiety this engenders in staff, staff dissatisfaction, and the concerns for the quality and safety of care being delivered during the digital transformation.^138^ Elements of digital disruption are seen in almost all of the barriers identified in this work. Facilitators, such as those that stipulate early staff involvement, staff training, and user championing, have been promoted for many years as mitigants for these barriers. If the issues raised by Sullivan and Staib^138^ as elements of digital disruption are still evident, it is possibly because the selected facilitators do not adequately deal with the barriers identified, or they were not successfully employed by the authors during their hospital's implementation project. Policymakers and clinicians still struggle with barriers that only serve to limit widespread acceptance and adoption of HIS.^139^, ^140^, ^141^

### 5.2 The legal position for eHealth technologies

It is common for the hospitals implementing HIS to not even be party to the contract.^142^ This is certainly the case when health departments and state organisations use *centrally negotiated contracts* (CNC).^142^ CNC use impacts communication, placing multiple layers of organisations between clinical user and developer.^142^ CNCs prevent HIS users from having proactive roles in negotiating terms in HIS vendor contracts,^26^ exacerbating issues when they do occur as vendors cannot always be expected to make decisions in the best interests of the delivery of health care.^26^

All health care procedures, tools, products, and services come with inherent risks, along with patient‐harboured expectations of the level of quality and the standards of care.^143^ Legislation on general liability in most countries makes reference to standards a patient may reasonably expect.^143^ The questions that are much harder to answer are as follows: whether a duty of care is owed when issues arise out of the use of EHR and LHS; and who owes that duty of care to the patient? While legislation and the common law *Bolam Test* in countries following the English legal tradition deal with situations where treating physicians breach a duty of care, it seems that no current legislation adequately contemplates or addresses general liability or duty of care issues arising from use of the multitude of eHealth products, from the seemingly simple EHR through to the multitude of complicated diagnostic medical devices, implantable technologies, software products, and prescribable mHealth apps.^143^, ^144^

### 5.3 Enabling LHS

The barriers identified by this study represent the substantive issues impeding implementation and adoption of LHS. However, few authors are asking the right questions, such as how can health departments achieve subject matter expertise in all technology, legal, compliance, and privacy aspects? Nor are they recognising that these issues must be resolved in order to achieve a secure data repository to support LHS.^3^, ^48^ The literature contains abundant discussion of requirements or elements of a solution to one or more of the barriers, mostly revolving around calls for a new and common set of standards.^48^, ^93^ However, we conclude that the absence of universal and effective LHS shows these barriers remain unresolved. While LHS have been developed that are intended to learn from evidence‐based literatures, patents, genomics, and other non‐patient data, LHS can only be successful in their ultimate goal of delivering ubiquitous *individualised* or *personalised* health care when data from EHR are made available. Data sharing to create large‐scale data warehouses will only occur when clinicians and patients can trust that methods and systems used are protecting their privacy. Even then, ethics review processes may still impede the realisation of knowledge that can come from LHS.

It became clear during this study that the majority of facilitation efforts is focused on human factors and more specifically the mitigation of negative aspects arising from a general resistance to change. More facilitators fell within the skills and management aspect of ITPOSMO‐BBF than any other: an aspect domain that primarily deals with staff, the skills they possess, whether these are sufficient to using the HIS being implemented, and the structure and style of management within the health care organisation.^9^, ^42^ While many of these facilitators should lead towards a smoother and more successful implementation, only one benefit was directly ascribed by authors to this aspect domain: staff retention. As a result, the greatest mitigation effort has been focused in an area that on review has least amount of tangible benefit. Other areas described with benefits bringing more significant impact on patient safety, health outcomes, and efficiency, such as those of the process and objectives aspects are left unresolved. Further research is needed to provide those implementing HIS with a more focused toolkit capable of mitigating a wider range of barriers and enabling delivery of the broadest possible benefits.

It is for those involved in developing HIS to actively participate in counteracting the barriers and changing negative perceptions. The barriers and issues for LHS identified in this research were largely similar to those previously ascribed to EHR, with the key additional issue that good quality EHRs are a necessity to enable LHS.^1^ Variations on *Meaningful Use* legislation seen in the United States, the United Kingdom, and Australia are aimed at supporting use of EHR in LHS, motivating expensive government‐operated national solutions like Care.data (UK), Shared Care Records (NZ), and MyHealth (AUS). While the cost to implement and maintain standardised EHR repositories in support of LHS may seem substantial, the cost savings promoted as justification for engaging LHS are potentially many times more significant.^64^

Many government, academic, and private organisations are funding research into novel health technologies aimed at realising the benefits identified by this study. A key theme within the facilitating factors for EHRs is clinician involvement, whether it be through early involvement or ongoing as HIS are integrated into the patient care environment. Many health technology implementations have lacked the input and involvement of appropriate stakeholder group members. Seeking input from all stakeholders who will impact and be impacted by the HIS is a significant factor in reducing resistance and increasing adoption of technology that has the potential to help many.^145^ While there has been a call for clinicians and their training organisations to engage with technologists, those working in the technology sector must be similarly called to seek clinician involvement.^145^, ^146^ Clinicians and technologists must work as co‐investigators and leaders in the research and implementation of health technologies. Engaging each other as leaders and stakeholders to influence HIS design and implementation.^146^, ^147^

While this paper starts from the premise that comparative review of the EHR implementation literature can provide a framework for analysis of LHS implementation, with the potential to increase the number of successful implementations, future work to extend this might also include comparison through analysis using other established quality improvement and change management frameworks. Such analysis was outside the scope of this particular work.

## 6 SUMMARY AND CONCLUSION

Prior to this report, there had been no study into the barriers, benefits, and facilitators for LHS implementation. Each of the EHR reviews used in this study discussed facilitators for successful implementation. We found that EHR and LHS share many similar barriers and facilitators and argue that some or all EHR benefits are relevant to and could be drawn from LHS. This may not be unexpected, as EHR are a key enabling technology for LHS, both are HIS, and LHS are poised to be as disruptive a technology to health care as EHR have been. We believe the primary goal in researching and designing new HIS is that they be used to make more precise diagnosis, select personalised treatment options, and improve overall health outcomes for patients. We argue that of all the potential facilitators discussed, ensuring the widest and most appropriate group of stakeholders, including patients, may be the most significant factor for ensuring success in the implementation of any new health technology.

Learning health systems have potential to be one of the major computing technological advances in health care. The objective of this paper was to present an analysis from an investigation of the barriers, benefits, and facilitating factors identified from the literature of EHR and LHS. This was undertaken in order to create a basis for discussion on how best to expedite successful implementation and adoption of LHS for clinical application. In the methodology for this paper, we used an extension of the ITPOSMO methodology, we termed ITPOSMO‐BBF. A key result is that although no prior research has analysed barriers and facilitators for LHS, EHR and LHS are seen to share similar barriers and facilitators. We also found that most facilitator effort in implementing both EHR and LHS involve addressing barriers that are best described as human factors, even though they carried the least number of author‐identified benefits. The process and objectives barriers that would appear to bring the greatest number of patient outcome, safety, and quality benefits remain unresolved, requiring significantly more attention in order to ensure the goals of LHS can be realised.

LHS envisage the constant generation of new clinical knowledge and operational insights based on the central role of clinical knowledge and collections of EHR. Once LHS are constructed and made operational, they can trigger new data streams of knowledge into the EHR, drawn from the analysis of thousands of prior patient interactions contrasted with evidence and experiential knowledge. In this vision, LHS and EHR have a symbiotic relationship. The implementation and adoption of EHRs have proved challenging for many organisations around the world, and there are many lessons arising from these challenges for LHS. This paper is unique in presenting a framework for, and a systematic analysis using, a new framework based on extending ITPOSMO with consideration of benefits and consolidation of overall knowledge relating to barriers, benefits, and facilitators. This framework simply and succinctly relates barriers to facilitators and aids those implementing LHS to understand where the significant or important benefits can be realised. ITPOSMO‐BBF will aid those implementing LHS to ensure their facilitation efforts can be focused in commensurate amounts to the degree of benefit that comes from resolving each set of barriers.

## CONFLICT OF INTEREST

No author identified a competing interest relevant to this research.

## AUTHOR CONTRIBUTIONS

S.M. performed the primary research and prepared the first draft. O.J. proposed the basis for the framework, refined by S.M. and K.D. Clinical input and review were provided by D.B. Model presentation was refined by H.P. and O.J. with editorial review and rewrites by K.D., O.J., T.G., and N.F. K.D. and N.F. supervised the research. All authors contributed, commented, and approved the final draft.

## References

[1] McLachlan S , Potts HWW , Dube K , et al. The Heimdall framework for supporting characterisation of learning health systems. BCS J Innov Health Inf. 2018;25(2). 10.14236/jhi.v25i2.996):77‐87. 30398449

[2] Friedman CP , Allee NJ , Delaney BC , et al. The science of learning health systems: foundations for a new journal. Learn Health Syst. 2017;1(1). 10.1002/lrh2.10020 PMC6516721 31245555

[3] Friedman C , Rubin J , Brown J , et al. Toward a science of learning systems: a research agenda for the high‐functioning learning health system. J Am Med Inform Assoc (JAMIA). 2015;22:43‐50. 25342177 10.1136/amiajnl-2014-002977 PMC4433378

[4] McLachlan S , Dube K , Buchanan D , et al. Learning health systems: the research community awareness challenge. BCS J Innov Health Inf. 2018;25(1). 10.14236/jhi.v25i1.981):038. 29717954

[5] McGinn CA , Grenier S , Duplantie J , et al. Comparison of user groups' perspectives of barriers and facilitators to implementing electronic health records: a systematic review. BMC Med. 2011;9(1):46. 21524315 10.1186/1741-7015-9-46 PMC3103434

[6] Jamoom EW , Patel V , Furukawa MF , King J . EHR adopters vs. non‐adopters: impacts of, barriers to, and federal initiatives for EHR adoption. Healthc Amst. 2014;2(1). 10.1016/j.hjdsi.2013.12.004):33‐39. 26250087 PMC4878018

[7] Tang PC , Ash JS , Bates DW , Overhage JM , Sands DZ . Personal health records: definitions, benefits and strategies for overcoming barriers to adoption. J Am Med Inform Assoc. 2006;13(2):121‐126. 16357345 10.1197/jamia.M2025 PMC1447551

[8] Friedman DJ , Parrish RG II . The population health record: concepts, definition, design and implementation. J Am Med Inform Assoc. 2010;17(4):359‐366. 20595299 10.1136/jamia.2009.001578 PMC2995645

[9] Heeks D , Mundy D , Salazar A . Why healthcare information systems succeed or fail In: Information Systems for Public Sector Management. Manchester, UK: Institute for Development Policy and Management; 1999.

[10] Pucihar, A. , Bogataj, K. , and Wimmer, M. . Gap analysis methodology for identifying future ICT related eGovernment research topics‐case of “Ontology and Semantic Web” in the context of eGovernment. in BLED 2007.2007.

[11] Hayrinen K , Saranto K , Nykanen P . Definition, structure, content, use and impacts of electronic health records: a review of the research literature. Int J Med Inform. 2008;77(5):291‐304. 17951106 10.1016/j.ijmedinf.2007.09.001

[12] Basole R , Braunstein M , Sun J . Data and analytics challenges for a learning healthcare system. ACM J Data Inf Qual. 2015;6(2).

[13] Foley TJ , Vale L . What role for learning health systems in quality improvement within healthcare providers? Learn Health Syst. 2017;1(4):e10025. 10.1002/lrh2.10025 PMC6508561 31245567

[14] Miller A , Moon B , Anders S , Walden R , Brown S , Montella D . Integrating computerised clinical decision support systems into clinical work: a meta‐synthesis of qualitative research. Int J Med Inform. 2015;84(12):1009‐1018. 26391601 10.1016/j.ijmedinf.2015.09.005

[15] Miriovsky BJ , Shulman L , Abernethy A . Importance of health information technology, electronic health records, and continuously agregating data to comparative effectiveness research and learning healthcare. J Clin Oncol. 2012;30(34):4243‐4248. 23071233 10.1200/JCO.2012.42.8011

[16] IoM , Digital infrastructure for the learning health system: the foundation for continuous improvement in health and health care. 2011, Institute of Medicine: https://www.ncbi.nlm.nih.gov/books/NBK83569/pdf/Bookshelf_NBK83569.pdf. 22379651

[17] Greene S , Reid R , Larson E . Implementing the learning health system: from concept to action. Ann Intern Med. 2012;157(3):207‐210. 22868839 10.7326/0003-4819-157-3-201208070-00012

[18] Angus D . Fusing randomised trials with big data: the key to self‐learning health care systems. J Am Med Assoc (JAMA). 2015;314(8):767‐768. 10.1001/jama.2015.7762 26305643

[19] Haynes B , Haines A . Barriers and bridges to evidence based clinical practice. Br Med J. 1998;317(7153):273‐276. 9677226 10.1136/bmj.317.7153.273 PMC1113594

[20] Casey JA , Schwartz BS , Stewart WF , Adler NE . Using electronic health records for population health research: a review of methods and applications. Annu Rev Public Health. 2016;37(1):61‐81. 10.1146/annurev-publhealth-032315-021353 26667605 PMC6724703

[21] Scott PJ , Curley PJ , Williams PB , Linehan IP , Shaha SH . Measuring the operational impact of digitized hospital records: a mixed methods study. BMC Med Inform Decis Mak. 2016;16(1):143. 27829453 10.1186/s12911-016-0380-6 PMC5103462

[22] Trotman J , Trinh J , Kwan YL , et al. Formalising multidisciplinary peer review: developing a haematological malignancy‐specific electronic proforma and standard operating procedure to facilitate procedural efficiency and evidence‐based clinical practice. Intern Med J. 2017;47(5):542‐548. 27753208 10.1111/imj.13302

[23] Johnson OA , Fraser HSF , Wyatt JC , Walley JD . Electronic health records in the UK and USA. Lancet. 2014;384(9947):954. 10.1016/S0140-6736(14)61626-3 25220971

[24] Greenhalgh T , Stramer K , Bratan T , Byrne E , Russell J , Potts HWW . Adoption and non‐adoption of a shared electronic summary record in England: a mixed‐method case study. Br Med J. 2010;340(jun16 4):c3111. 20554687 10.1136/bmj.c3111

[25] HOCCOPA . The dismantled National Programme for IT in the NHS. London, UK: House of Commons Committee of Public Accounts; 2013.

[26] Koppel R , Kreda D . Healthcare IT usability and sustainability for clinical needs: challenges of design, workflow, and contractual relations. Stud Health Technol Inform. 2010;157:7‐14. 20543360

[27] Greenhalgh T , Potts HWW , Wong G , Bark P , Swinglehurst D . Tensions and paradoxes in electronic patient record research: a systematic literature review using the meta‐narrative method. Milbank Q. 2009;87(4):729‐788. 10.1111/j.1468-0009.2009.00578.x 20021585 PMC2888022

[28] Sheikh A , Cornford T , Barber N , et al. Implementation and adoption of nationwide electronic health records in secondary care in England: final qualitative results from prospective national evaluation in “early adopter” hospitals. Br Med J. 2011;343(oct17 1):d6054. 22006942 10.1136/bmj.d6054 PMC3195310

[29] Topaz, M. , Ronquillo C , Peltonen LM , Pruinelli L , Sarmiento RF , Badger MK , Ali S , Lewis A , Georgsson M , Jeon E , Tayaben JL , Kuo CH , Islam T , Sommer J , Jung H , Eler GJ , Alhuwail D , Lee YL , Nurse informaticians report low satisfaction and multi‐level concerns with electronic health records: results from an international survey, in AMIA Annual Symposium Proceedings. 2016. PMC5333337 28269961

[30] Imison C , Castle‐Clarke S , Watson R , Edwards N . Delivering the Benefits of Digital Health Care. Nuffield Trust; 2016.

[31] Linder JA , Ma J , Bates DW , Middleton B , Stafford RS . Electronic health record use and the quality of ambulatory care in the United States. Arch Intern Med. 2007;167(13):1400‐1405. 17620534 10.1001/archinte.167.13.1400

[32] Cresswell K , Worth A , Sheikh A . Integration of a nationally procured electronic health record system into user work practices. BMC Med Inform Decis Mak. 2012;12(1). 10.1186/1472-6947-12-15 PMC3313868 22400978

[33] Liyanage H , Correa ST , Kuziemsky C , Terry AL , de Lusignan S . Does informatics enable or inhibit the delivery of patient‐centred, coordinated, and quality‐assured care: a Delphi study. IMIA Yearb Med Inform. 2015;10(1). 10.15265/IY-2015-017 PMC4587041 26123905

[34] Tolar M , Balka E . Caring for individual patients and beyond: enhancing care through secondary use of data in a general practice setting. Int J Med Inform. 2012;81(7):461‐474. 22285076 10.1016/j.ijmedinf.2012.01.003

[35] Protti D . Missed connections: The adoption of information technology in Canadian healthcare. CD Howe Inst. 2015;422.

[36] Russo E , Sittig DF , Murphy DR , Singh H . Challenges in patient safety improvement research in the era of electronic health records. Health. 2016;4(4):285‐290. 10.1016/j.hjdsi.2016.06.005 27473472

[37] Adler‐Milstein J , Daniel G , Grossmann C , et al. Return on information: a standard model for assessing institutional return on electronic health records. Inst Med Natl Acad. 2014;1‐21.

[38] Perlin J . Health information technology interoperability and use for better care and evidence. J Am Med Assoc. 2016;316(16):1667‐1668. 10.1001/jama.2016.12337 27669026

[39] Yao, W. , Chu, C. , and Li, Z. , The Use of RFID in Healthcare: Benefits and Barriers, in 2010 IEEE International Conference on RFID‐Technology and Applications (RFID‐TA). 2010, IEEE p. 128‐134.

[40] Vaismoradi M , Turunen H , Bondas T . Content analysis and thematic analysis: implications for conducting a qualitative descriptive study. Nurs Health Sci. 2013;15(3):398‐405. 23480423 10.1111/nhs.12048

[41] Stumme G . Formal concept analysis In: Handbook on Ontologies. Vol.177‐199 Berlin: Springer; 2009.

[42] Syamsuddin I . Novel gap analysis framework for cloud health information systems. J Theor Appl Inf Technol. 2016;87(3):415‐421.

[43] Rugchatjaroen K . Success of electronic government project in Bangkok Metropolis: an ITPOSMO approach. Int J Soc Sci Humanit Stud. 2015;5(9):783‐787.

[44] Maarten H . A modern Socrates discourse in a local e‐government setting. Futur E‐Gov Learn Past. 2016;4(3):1‐12.

[45] Hassan H , Shehab E , Peppard J . Toward full public e‐service environment in developing countries. World Acad Sci Eng Technol. 2010;4(6):618‐622.

[46] Musen M , Blackford M , Greenes R . Clinical decision‐support systems In: Biomedical Informatics. London: Springer; 2014:643‐674.

[47] Curcin V . Embedding data provenance into the learning health system to facilitate reproducible research. Learn Health Syst. 2017;1(2):e10019. 10.1002/lrh2.10019 PMC6516719 31245557

[48] Davidson A . Creating value: unifying silos into public health business intelligence. eGEMS. 2015;2(4). 10.13063/2327-9214.1172 PMC4438104 25995989

[49] Feeley TW , Sledge GW , Levit L , Ganz PA . Improving the quality of cancer care in America through health information technology. J Am Med Inform Assoc. 2014;21(5):772‐775. 24352553 10.1136/amiajnl-2013-002346 PMC4147622

[50] Furukawa MF , King J , Patel V , Hsiao CJ , Adler‐Milstein J , Jha AK . Despite substantial progress in EHR adoption, health information exchange and patient engagement remain low in office settings. Health Aff. 2014;33(9):1672‐1679. 10.1377/hlthaff.2014.0445 25104827

[51] Jameson J , Longo D . Precision medicine—personalized, problematic and promising. N Engl J Med. 2015;372(23):2229‐2234. 26014593 10.1056/NEJMsb1503104

[52] Krumholz H . Big data and new knowledge in medicine: the thinking, training and tools needed for a learning health system. Health Aff. 2014;33(7):1163‐1170. 10.1377/hlthaff.2014.0053 PMC5459394 25006142

[53] Kaye R , Kokia E , Shalev V , Idar D , Chinitz D . Barriers and success factors in health information technology: a practitioners perspective. J Manag Mark Healthc. 2010;3(2):163‐175.

[54] Nguyen L , Bellucci E , Nguyen L . Electronic health records implementation: an evaluation of information system impact and contingency factors. Int J Med Inform. 2014;83(11):779‐796. 25085286 10.1016/j.ijmedinf.2014.06.011

[55] Chao WC , Hu H , Ung COL , Cai Y . Benefits and challenges of electronic health record system on stakeholders: a qualitative study of outpatient physicians. J Med Syst. 2013;37(4):9960. 23852368 10.1007/s10916-013-9960-5

[56] Houser S , Johnson L . Perceptions regarding electronic health record implementation among health information management professionals in Alabama: a statewide survey and analysis. Perspect Health Inf Manag. 2008;5(6). PMC2394577 18504505

[57] Abernethy AP , Ahmad A , Zafar SY , Wheeler JL , Reese JB , Lyerly HK . Electronic patient‐reported data capture as a foundation of rapid learning cancer care. Med Care. 2010;48(6):S32‐S38. 20473201 10.1097/MLR.0b013e3181db53a4

[58] Bhandari RP , Feinstein AB , Huestis SE , et al. Pediatric‐Collaborative Health Outcomes Information Registry (Peds‐CHOIR): a learning health system to guide pediatric pain research and treatment. Pain. 2017;157(9):2033‐2044. 10.1097/j.pain.0000000000000609 PMC4988911 27280328

[59] Braithwaite S , Stine N . Health‐weighted composite quality metrics offer promise to improve health outcomes in a learning health system. eGEMS. 2013;1(2). 10.13063/2327-9214.1022 PMC4371421 25848572

[60] Friedman C , Wong A , Blumenthal D . Achieving a nationwide learning health system. Sci Transl Med. 2010;2(57):1‐3. 10.1126/scitranslmed.3001456 21068440

[61] Gold M , Hossain M , Mangum A . Consumer engagement in health IT: distinguishing rhetoric from reality. eGEMS. 2015;3(1). 10.13063/2327-9214.1190 PMC4672873 26665120

[62] Bernstein JA , Friedman C , Jacobson P , Rubin JC . Ensuring public health's future in a national‐scale learning health system. Am J Prev Med. 2015;48(4):480‐487. 25700654 10.1016/j.amepre.2014.11.013

[63] Bellack J . Creating a continuously learning health system through technology: a call to action. J Nurs Educ. 2016;55(1):3‐5. 26812374 10.3928/01484834-20151214-01

[64] Morain S , Kass N , Grossman C . What allows a health care system to become a learning health care system: results from interviews with health system leaders. Learn Health Syst. 2016;1(1). 10.1002/lrh2.10015 PMC6516720 31245552

[65] Turley C . Leveraging a statewide clinical data warehouse to expand boundaries of the learning health system. eGEMS. 2016;4(1). 10.13063/2327-9214.1245 PMC5226381 28154834

[66] Koppel R , Metlay JP , Cohen A , et al. Role of computerized physician order entry systems in facilitating medication errors. J Am Med Assoc (JAMA). 2005;293(10):1197‐1203. 10.1001/jama.293.10.1197 15755942

[67] Menachemi N , Collum T . Benefits and drawbacks of electronic health record systems. Risk Manage Healthc Pol. 2011;4:47‐55. 10.2147/RMHP.S12985 PMC3270933 22312227

[68] Azar J , Adams N , Boustani M . The Indiana University Center for healthcare innovation and implementation science: bridging healthcare research and delivery to build a learning healthcare system. Z Evid Fortbild Qual Gesundhwes. 2015;109(2):138‐143. 26028451 10.1016/j.zefq.2015.03.006

[69] Wysham N , Howie L , Patel K , Cameron C . Development and refinement of a learning health systems training program. eGEMS. 2016;4(1). 10.13063/2327-9214.1236 PMC5226386 28154832

[70] Cherry B , Carter M , Owen D , Lockhart C . Factors affecting electronic health record adoption in long‐term care facilities. J Healthc Qual. 2008;30(2):37‐47. 10.1111/j.1945-1474.2008.tb01133.x 18411891

[71] Lluch M . Healthcare professionals' organisational barriers to health information technologies: a literature review. Int J Med Inform. 2011;80(12):849‐862. 22000677 10.1016/j.ijmedinf.2011.09.005

[72] Terry A , Thorpe CF , Giles G , et al. Implementing electronic health records. Can Fam Physician. 2008;54. PMC2377228 18474707

[73] Schumaker R , Reganti K . Implementation of electronic health record (EHR) system in the healthcare industry. Int J Priv Health Inf Manag. 2014;2(2):57‐71.

[74] Cresswell K , Mozaffar H , Lee L , Williams R , Sheikh A . Safety risks associated with the lack of integration and interfacing of hospital health information technologies: a qualitative study of hospital electronic prescribing systems in England. Br Med J Qual Saf. 2016;0:1‐12. 10.1136/bmjqs-2015-004925 27037303

[75] Corrigan D , Munnelly G , Kazienko P , et al. Requirements and validation of a prototype learning health system for clinical diagnosis. Learn Health Syst. 2017;1(4):e10026. 10.1002/lrh2.10026 PMC6508515 31245568

[76] Daniel C , Ouagne D , Sadou E , et al. Cross border semantic interoperability for learning health systems: the EHR4CR semantic resources and services. Learn Health Syst. 2015;1(1):e10014 10.1002/lrh2.10014. PMC6516724 31245551

[77] Kuchinke W , Ohmann C , Verheij RA , van Veen E , Delaney B . Development towards a learning health system—experiences with the privacy protection model of the TRANSFoRm project In: Data Protection on the Move. Netherlands: Springer; 2016:101‐134.

[78] Weng C , Li Y , Berhe S , et al. An integrated model for patient care and clinical trials (IMPACT) to support clinical research visit scheduling workflow for future learning systems. J Biomed Inform. 2013;46(4):642‐652. 23684593 10.1016/j.jbi.2013.05.001 PMC3716847

[79] Coiera E . The forgetting health system. Learn Health Syst. 2017;2. 10.1002/lrh2.10023 PMC6508563 31245565

[80] Steinwachs D . Transforming public health systems: using data to drive organizational capacity for quality improvement and efficiency. eGEMS. 2015;2(4). 10.13063/2327-9214.1175 PMC4438105 25995990

[81] Schneeweiss S . Learning from big health care data. N Engl J Med. 2014;370(23):2161‐2163. 24897079 10.1056/NEJMp1401111

[82] Khurshid A . A tale of two cities: developing health information platforms for a learning health system in Austin and in New Orleans. Learn Health Syst. 2017;1(2):e10017.

[83] Mason A , Barton A . The emergence of a learning healthcare system. Clin Nurse Spec. 2013;27(1):7‐9. 23222020 10.1097/NUR.0b013e3182776dcb

[84] Everson J . The implications and impact of 3 approaches to health information exchange: community, enterprise, and vendor‐mediated health information exchange. Learn Health Syst. 2016;1(2)e10021):1‐9. 10.1002/lrh2.10021 PMC6508570 31245558

[85] Crawford LS , Matczak GJ , Moore EM , Haydar RA , Coderre PT . Patient‐centered drug development and the learning health system. Learn Health Syst. 2017 e10027;1(3):1‐7. 10.1002/lrh2.10027. PMC6508534 31245560

[86] Winden, T. , Chen, E. , and Melton, G. . Representing residence, living situation, and living conditions: an evaluation of terminologies, standards, guidelines, and measures/surveys. In 2016 Conference of the American Medical Informatics Association. 2016. American Medical Informatics Association PMC5333311 28269967

[87] Doebbeling B , Flanagan M . Emerging perspectives on transforming the healthcare system: redesign strategies and a call for needed research. Med Care. 2011;49(12):s59‐s64. 22095034 10.1097/MLR.0b013e31821b57eb

[88] Starren J , Winter A , Lloyd‐Jones D . Enabling a learning health system through a unified enterprise data warehouse: the experience of the Northwestern University Clinical and Translational Sciences (NUCATS) Institute. Clin Transl Sci J. 2015;8(4):269‐271. 10.1111/cts.12294 PMC4553136 26032246

[89] Tuot DS , Leeds K , Murphy EJ , et al. Facilitators and barriers to implementing electronic referral and/or consultation systems: a qualitative study of 16 health organisations. BMC Health Serv Res. 2015;15 10.1186/s12913-015-1233-1(1):568. 26687507 PMC4684927

[90] Lee S , Kelley M , Cho M , et al. Adrift in the gray zone: IRB perspectives on research in the learning health system. AJOB Empir Bioeth. 2016;7(2):125‐134. 27917391 10.1080/23294515.2016.1155674 PMC5130156

[91] Etheredge L . Rapid learning: a breakthrough agenda. Health Aff. 2014;33(7):1155‐1162. 10.1377/hlthaff.2014.0043 25006141

[92] Deeny S , Steventon A . Making sense of the shadows: priorities for creating a learning healthcare system based on routinely collected data. BMJ Qual Saf. 2015;24(8):505‐515. 10.1136/bmjqs-2015-004278 PMC4515981 26065466

[93] Etheredge L . A rapid‐learning health system. Health Aff. 2007;26(2):107‐118. 10.1377/hlthaff.26.2.w107 17259191

[94] Gardner W . Policy capacity in the learning healthcare system. Int J Health Pol Manag. 2015;4(12):841‐843. 10.15171/ijhpm.2015.147 PMC4663089 26673470

[95] Wilk A , Platt J . Measuring physicians' trust: a scoping review with implications for public policy. Soc Sci Med. 2016;165:75‐81. 27497858 10.1016/j.socscimed.2016.07.039

[96] Rumbold J , Pierscionek B . A critique of the regulation of data science in healthcare research in the European Union. BMC Med Ethics. 2017;18(1):27. 28388916 10.1186/s12910-017-0184-y PMC5385067

[97] Krumholz H , Terry S , Waldstreicher J . Data acquisition, curation and use for a continuously learning health system. J Am Med Assoc (JAMA). 2016;316(16):1669‐1670. 10.1001/jama.2016.12537 27668668

[98] Wiley . Learning Health Systems. 2017 13 September 2017]; Available from: http://onlinelibrary.wiley.com/journal/10.1002/(ISSN)2379‐6146.

[99] Wallace PJ , Shah ND , Dennen T , Bleicher PA , Crown WH . Optum labs: building a novel node in the learning health care system. Health Aff. 2014;33(7):1187‐1194. 10.1377/hlthaff.2014.0038 25006145

[100] Wong G , Greenhalgh T , Westhorp G , Buckingham J , Pawson R . RAMESES publication standards: realist syntheses. BMC Med. 2013;11(1):21 10.1186/1741-7015-11-21 23360677 PMC3558331

[101] Marsolo K , Margolis PA , Forrest CB , Colletti RB , Hutton JJ . A digital architecture for a network‐based learning health system—integrating chronic care management, quality improvement and research. eGEMS. 2015;3(1). 10.13063/2327-9214.1168 PMC4562738 26357665

[102] Mirnezami R , Nicholson J , Darzi A . Preparing for precision medicine. N Engl J Med. 2012;366(6):489‐491. 22256780 10.1056/NEJMp1114866

[103] Cresswell KM , Mozaffar H , Lee L , Williams R , Sheikh A . Safety risks associated with the lack of integration and interfacing of hospital health information technologies: a qualitative study of hospital electronic prescribing systems in England. BMJ Qual Saf. 2017;26(7):530‐541. 10.1136/bmjqs-2015-004925 27037303

[104] Lowes LP , Noritz GH , Newmeyer A , et al. ‘Learn from every patient’: implementation and early results of a learning health system. Dev Med Child Neurol. 2016;59(2):183‐191. 10.1111/dmcn.13227. 27545839

[105] Mandl KD , Kohane IS , McFadden D , et al. Scalable collaborative infrastruture for a learning healthcare system (SCILHS): architecture. J Am Med Inform Assoc. 2014;21(4):615‐620. 24821734 10.1136/amiajnl-2014-002727 PMC4078286

[106] Nwaru BI , Friedman C , Halamka J , Sheikh A . Can learning health systems help organisations deliver personalised care? BMC Med. 2017;15(1):177. 28965492 10.1186/s12916-017-0935-0 PMC5623976

[107] Rouse W , Johns M , Pepe K . Learning in the health care enterprise. Learn Health Syst. 2017;1(4):e10024. 10.1002/lrh2.10024 PMC6508503 31245566

[108] Yu, P. , Artz, D. , and Warner, J. , Electronic health records (EHRs): supporting ASCO's vision of cancer care. Electronic Health Records, 2014. ASCO Educational Book(2014). 10.14694/EdBook_AM.2014.34.225 24857080

[109] Ludwick D , Doucette J . Adopting electronic medical records in primary care: lessons learned from health information systems implementation experience in seven countries. Int J Med Inform. 2009;78(1):22‐31. 18644745 10.1016/j.ijmedinf.2008.06.005

[110] Jha AK , DesRoches CM , Campbell EG , et al. Use of electronic records in U.S. hospitals. N Engl J Med. 2009;360(16):1628‐1638. 19321858 10.1056/NEJMsa0900592

[111] Boonstra A , Versluis A , Vos J . Implementing electronic health records in hospitals: a systematic literature review. BMC Health Serv Res. 2014;14(1):370. 25190184 10.1186/1472-6963-14-370 PMC4162964

[112] Flores M , Glusman G , Brogaard K , Price ND , Hood L . P4 medicine: how systems medicine will transform the healthcare sector and society. Pers Med. 2013;10(6):565‐576. 10.2217/PME.13.57 PMC4204402 25342952

[113] Faden, R. , et al., An ethics framework for a learning health care system: a departure from traditional research ethics and clinical ethics., in Ethical Oversight of Learning Health Care Systems, Hastings Centre Report Special Report. 2013. 10.1002/hast.134 23315888

[114] Yu P , Hoffman M , Hayes D . Biomarkers and oncology. Arch Pathol Lab Med. 2015;139(4):451‐456. 25152310 10.5858/arpa.2014-0080-ED

[115] Boonstra A , Broekhuis M . Barriers to the acceptance of electronic medical records by physicians from systematic review to taxonomy and interventions. BMC Health Serv Res. 2010;10(1). 10.1186/1472-6963-10-231 PMC2924334 20691097

[116] Brody H , Miller F . The research‐clinical practice distinction, learning health system and relationships. Hastings Cent Rep. 2013;43(5):41‐47. 10.1002/hast.199 24092591

[117] Kelley M , James C , Alessi S , et al. Patient perspectives on the learning health system: the importance of trust and shared decision making. Am J Bioeth. 2015;15(9):4‐17. 10.1080/15265161.2015.1062163 PMC4821628 26305741

[118] Roth M , Rubin JC , Omollo K , Friedman CP , Seagull FJ . The learning health‐system: a new frontier for human factors In: 2016 International Symposium on Human Factors and Ergonomics in Health Care: Improving the Outcomes. Human Factors and Ergonomics Society; 2016.

[119] Pletcher M , Lo B , Grady D . Informed consent in randomised quality improvement trials: a clinical barrier for learning health systems. J Am Med Assoc (JAMA). 2014;174(5):668‐670. 10.1001/jamainternmed.2013.13297 24615554

[120] McNolty L , Payne R . Relying on trust for research on medical practice in learning health systems. Am J Bioeth. 2015;15(9):30‐32. 10.1080/15265161.2015.1062172 26305748

[121] Jawhari B , Ludwick D , Keenan L , Zakus D , Hayward R . Benefits and challenges of EMR implementations in low resource settings: a state‐of‐the‐art review. BMC Med Inform Decis Mak. 2016;16(1):116. 27600269 10.1186/s12911-016-0354-8 PMC5011989

[122] Friedman C , Rigby M . Conceptualising and creating a global learning health system. Int J Med Inform. 2013;82(4):e63‐e71. 22717661 10.1016/j.ijmedinf.2012.05.010

[123] Pare G , Raymond L , de Guinea AO , et al. Barriers to organizational adoption of EMR systems in family physician practices: a mixed‐methods study in Canada. Int J Med Inform. 2014;83(8):548‐558. 24969270 10.1016/j.ijmedinf.2014.06.003

[124] Kwon S , Florence M , Grigas P , et al. Creating a learning healthcare system in surgery: Washington State's Surgical Care and Outcomes Assessment Program (SCOAP) at 5 years. Surgery. 2011;151(2). 10.1016/j.surg.2011.08.015):146‐152. 22129638 PMC4208432

[125] Zandieh SO , Yoon‐Flannery K , Kuperman GJ , Langsam DJ , Hyman D , Kaushal R . Challenges to EHR implementation in electronic‐ versus paper‐based office practices. J Gen Intern Med. 2008;23(6):755‐761. 18369679 10.1007/s11606-008-0573-5 PMC2517887

[126] Amin W , Tsui F , Borromeo C , et al. PaTH: towards a learning health system in the Mid‐Atlantic region. J Am Med Inform Assoc (JAMIA). 2014;21(4):633‐636. 24821745 10.1136/amiajnl-2014-002759 PMC4078296

[127] Kumar S , Hanss T , Johnson L , et al. Leveraging contextual inquiry methods to empower patients in a learning health system In: 48th International Conference on System Sciences. Hawaii: IEEE; 2015.

[128] Rubin J . Patient empowerment and the learning health system. Learn Health Syst. 2017;1(3):e10030. 10.1002/lrh2.10030 PMC6508488 31245562

[129] Rubin J , Friedman C . Weaving together a healthcare improvement tapestry: learning health system brings together health IT data stakeholders to share knowledge and improve health. J AHIMA. 2014;85(5):38‐43. 24938034

[130] Scheuner MT , de Vries H , Kim B , Meili RC , Olmstead SH , Teleki S . Are electronic health records ready for genomic medicine? Genet Med. 2009;11(7):510‐517. 19478682 10.1097/GIM.0b013e3181a53331

[131] Forrest CB , Margolis P , Seid M , Colletti RB . PEDSnet: how a prototype pediatric learning health system is being expanded into a national network. Health Aff. 2014;33(7):1171‐1177. 10.1377/hlthaff.2014.0127 25006143

[132] Cresswell K , Sheikh A . Organizational issues in the implementation and adoption of health information technology innovations: an interpretative review. Int J Med Inform. 2013;82(5):e73‐e86. 23146626 10.1016/j.ijmedinf.2012.10.007

[133] MelbUni . Graduate certificate in health informatics and digital health. 2017 [cited 2018 17 May]; Available from: https://mdhs‐study.unimelb.edu.au/degrees/graduate‐certificate‐in‐health‐informatics‐and‐digital‐health/overview.

[134] UTAS . Introduction to health informatics. 2017 [cited 2018 17 May]; Available from: http://www.utas.edu.au/courses/chm/units/crh500‐introduction‐to‐health‐informatics.

[135] Lea A , Pearson D , Clamp S , Johnson O , Jones R . Undergraduate learning: using the electronic medical record within medical undergraduate education. Educ Prim Care. 2008;19(6):656‐659.

[136] Sugarman J , Calif R . Ethics and regulatory complexities for pragmatic clinical trials. J Am Med Assoc. 2014;311(23):2381‐2382. 10.1001/jama.2014.4164 24810723

[137] Reimer, K. and Hamann, J. . Digital disruption intermediaries. 2015 [cited 2018 17 May]; Available from: https://ses.library.usyd.edu.au/bitstream/2123/12761/7/ADTL_Digital%20Disruptive%20Intermediaries‐final.pdf.

[138] Sullivan C , Staib A . Digital disruption ‘syndromes’ in a hospital: important considerations for the quality and safety of patient care during rapid digital transformations. Aust Health Rev. 2018;42:294‐298. 28514640 10.1071/AH16294

[139] Kowitlawakul Y , Chan SWC , Pulcini J , Wang W . Factors influencing nursing students' acceptance of electronic health records for nursing education (EHRNE) software program. Nurse Educ Today. 2016;35(1):189‐194. 10.1016/j.nedt.2014.05.010 24947068

[140] Rathert C , Porter TH , Mittler JN , Fleig‐Palmer M . Seven years after meaningful use: physicians' and nurses' experiences with electronic health records. Health Care Manage Rev. 2017;1. 28614166 10.1097/HMR.0000000000000168

[141] Nicholls JA , Potts HWW , Coleman B , Patterson DL . Legal and professional implications of shared care: a case study in oral anticoagulation stroke prevention therapy. BMC Health Serv Res. 2015;15(1):93. 25889353 10.1186/s12913-015-0756-9 PMC4357224

[142] Robertson A , Cresswell K , Takian A , et al. Implementation and adoption of nationwide electronic health records in secondary care in England: qualitative analysis of interim results from a prospective national evaluation. Br Med J. 2010;341(sep01 3):c4564. 20813822 10.1136/bmj.c4564 PMC2933355

[143] Andoulsi I , Wilson P . Understanding liability in eHealth: towards greater clarity at European Union level In: George C, Whitehouse D, Duquenoy P, eds. eHealth: Legal, Ethical and Governance Challenges. Heidelberg, Berlin: Springer; 2013:165‐180.

[144] Byambasuren O , Sanders S , Beller E , Glasziou P . Prescribable mHealth apps identified from an overview of systematic reviews. Digit Med. 2018;1(1). 10.1038/s41746-018-0021-9 PMC6550270 31304297

[145] Chey, W. and Speigel, B. , The digital doctor: how technologies enhance health care, in Helio Gastroenterology. 2016.

[146] Clemensen J , Larsen SB , Kyng M , Kirkevold M . Participatory design in health sciences: using cooperative experimental methods in developing health services and computer technology. Qual Health Res. 2007;17(1):122‐130. 17170250 10.1177/1049732306293664

[147] Dickson, H. and Ham, C. , Engaging doctors in leadership: review of the literature. 2008, University Of Birmingham

